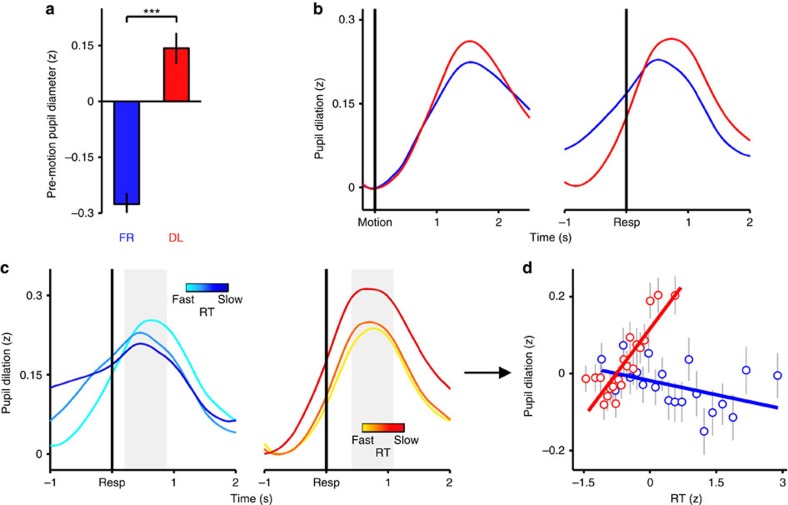# Erratum: Global gain modulation generates time-dependent urgency during perceptual choice in humans

**DOI:** 10.1038/ncomms14299

**Published:** 2017-01-18

**Authors:** Peter R Murphy, Evert Boonstra, Sander Nieuwenhuis

Nature Communications 7: Article number: 13526; DOI: 10.1038/ncomms13526 (2016); Published: 11 24 2016; Updated: 01 18 2017

In Fig. 4c of this Article, the colour bars were inadvertently changed from graded to solid during the production process. The correct version of Fig. 4c appears below as Fig. 1.

## Figures and Tables

**Figure 1:**