# Supplementary material for: Global gain modulation generates time-dependent urgency during perceptual choice in humans
Source: Nat Commun. 2016 Nov 24;7:13526. doi: 10.1038/ncomms13526 (PMC5123079; doi:10.1038/ncomms13526)
Supplement: Supplementary Information — Supplementary Figures 1 - 7, Supplementary Tables 1 and 2, Supplementary Methods and Supplementary References [file ncomms13526-s1.pdf]

Supplementary Figures

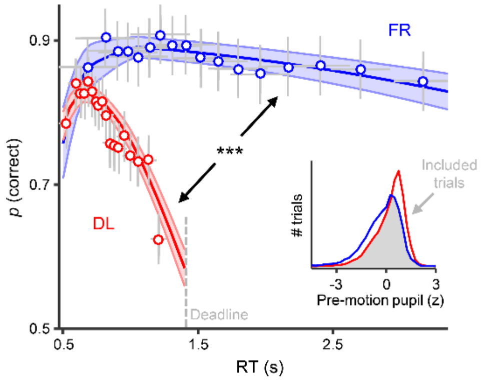

Supplementary Figure 1 | Conditional accuracy functions after matching trials for pre-motion pupil size. Points indicate mean accuracy of trials sorted by RT into 18 equal-sized bins and colored lines show best fits of piece-wise logistic regressions to each subject's single-trial data. Error bars and shaded areas indicate ±s.e.m. of data points and regression lines, respectively. The trial matching procedure consisted of discretizing each subject's pre-motion pupil distributions into bins of 0.1z width and, for each bin in which both conditions had at least one trial, randomly selecting without replacement a matched number of trials from the condition that had a greater trial count in that bin (see inset for representation of trial selection). CAFs were then calculated using selected trials across all bins, the matching procedure was repeated 100 times, and the final per-subject pupil-matched CAFs were calculated by averaging over all iterations. The strong effect of speed emphasis on the negative slope of the CAF is present after pupil-matching (*** = $p<0.001$). Notably, unlike for the cohort described in the main manuscript (Fig. 1c), estimated accuracy at deadline is greater than chance (58.3±2.5%; one-sample t-test with H_0=50%: t_{22}=3.4, p=0.003). This discrepancy may reflect weaker time-dependency in the cohort for this study (possibly due to differences in the temporal order of task block administration); alternatively, it may be due to the lower trial numbers (307±11 per condition) available for this analysis, which will produce less reliable CAF estimates particularly at the time of the deadline.

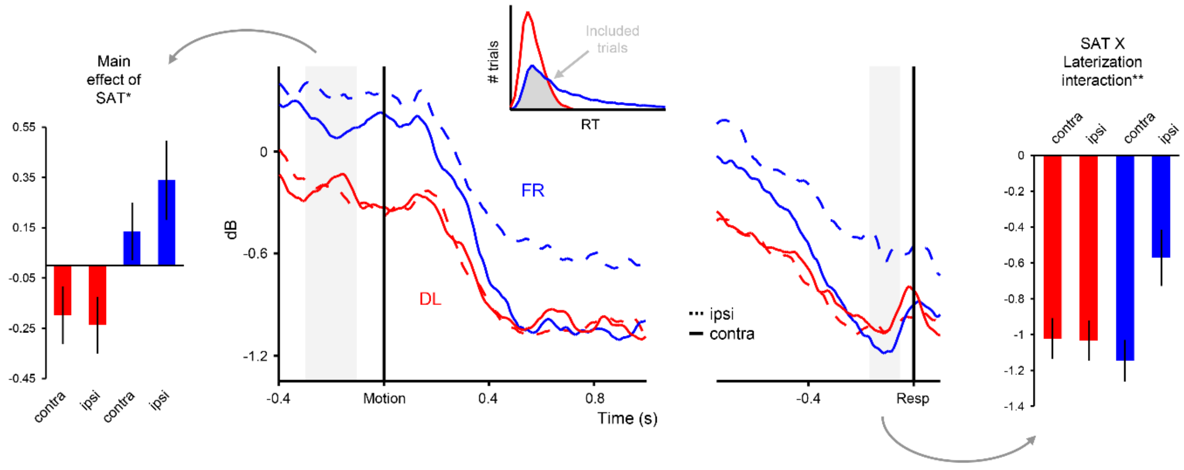

Supplementary Figure 2 | Effects of speed emphasis on motor-related 8-14Hz power after RT matching. The matching procedure consisted of discretizing each subject's RT distributions into bins of 10ms width and, for each bin in which both conditions had at least one trial, randomly selecting without replacement a matched number of trials from the condition that had a greater trial count in that bin (see inset for representation of trial selection). Averaged signals were then calculated using selected trials across all bins, the matching procedure was repeated 100 times, and the final per-subject RT-matched signals were calculated by averaging over all iterations. The pre-motion effect of speed regime (left), the similar level of contra-lateral pre-response desynchronization, and the lateralization*speed emphasis interaction on pre-response desynchronization (both right) were all still present after RT matching. Error bars indicate s.e.m. ** = $p<0.01$, * = $p<0.05$.

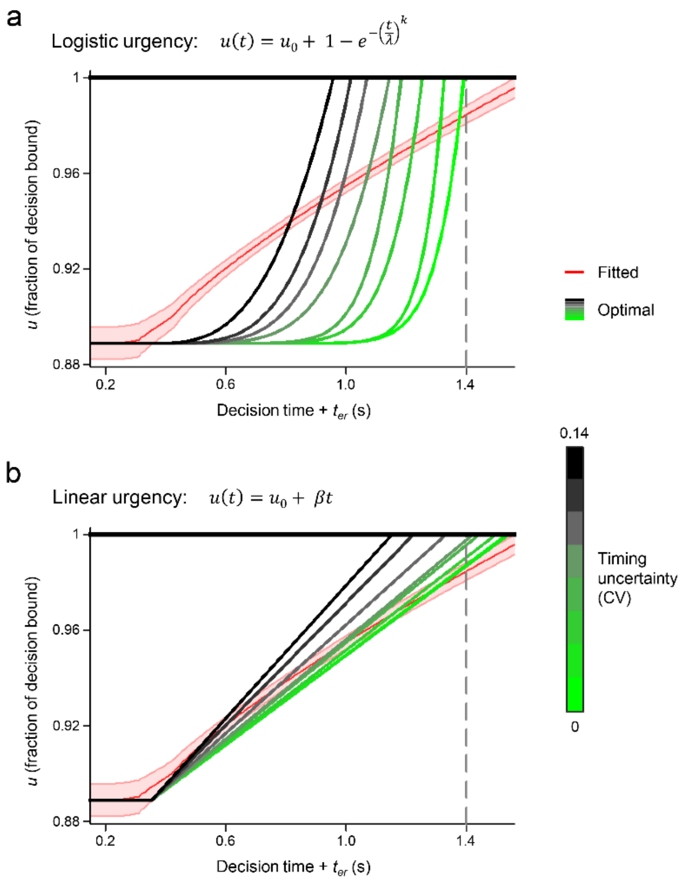

Supplementary Figure 3 | Optimal diffusion model urgency signals for two closed-form functions. (a) Optimal (reward-maximizing) additive urgency signals, parameterized according to the closed-form logistic function shown, for a fixed, representative combination of time-invariant DDM parameters (v = 0.112, η = 0.063, t_{er} = 0.355, u_0 = 0.889). Green (black) reflects low (high) levels of endogenous uncertainty over the timing of the deadline. With higher uncertainty, the deflection of the optimal urgency signal toward the decision bound (thick black horizontal line) occurs earlier, as does the point at which the urgency signal crosses the bound. This pattern is due to the extreme monetary punishment for missed deadlines relative to the payoffs for correct/incorrect decisions, which produces an optimal policy under uncertainty of foregoing longer and more accurate decisions to ensure that a response is always executed prior to the true deadline. The fitted urgency signals from the best-fitting urgency DDM are shown in red. Shaded region indicates s.e.m. (b) Same as in (a), but reward-maximizing urgency signals are now constrained to take the form of the simple linear function shown. When uncertainty is low, the optimal urgency signals do not fully reach the decision bound by the time of the deadline; this is because the penalties associated with the very few missed deadlines (<0.1% of all trials) under such an urgency regime are offset by the greater reward gleaned from the shallower urgency signal prior to the deadline. Note the similarity between the fitted urgency signals and the optimal cases for moderate-to-low levels of timing uncertainty. In both plots, timing uncertainty is quantified as the coefficient of variation (CV = SD/mean) of a normal distribution of subjective deadlines with a mean centered on the true deadline of 1.4s.

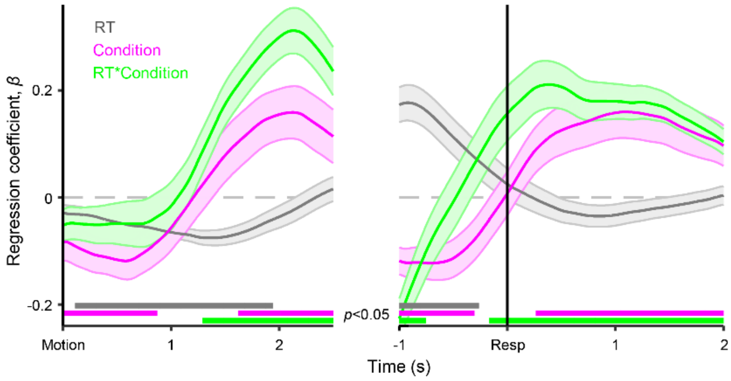

Supplementary Figure 4 | Temporal evolution of pupil dilation effects. Plots depict group-average regression coefficients from the single-trial linear regression model described in Supplementary Equation (6) and fit on a sample-by-sample basis across the entire stimulus- and response-aligned pupil dilation waveforms. This model includes terms for the effects on pupil dilation of response time (black), speed emphasis regime (pink) and their interaction (green). Shaded regions indicate s.e.m., and thick lines at bottom of each plot indicate time points at which each of the terms in the model are significantly different from zero ($p<0.05$, uncorrected).

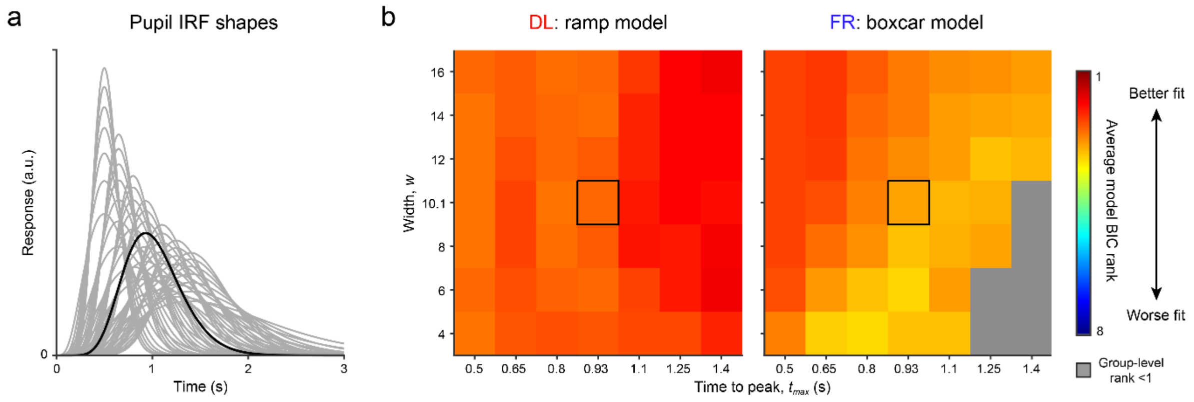

Supplementary Figure 5 | Pupil modelling effects are robust to different shapes of pupil IRF. (a) Shapes of the impulse response functions (IRFs) used for (b), with the canonical parameters (employed for the analysis depicted in Fig. 5 of the main text) plotted in black. The width (*w*) and time-to-peak (t_{max}) parameters are varied across the following ranges: *w*, 4, 6, 8, 10.1 (canonical), 12, 14, 16; t_{max}, 500, 650, 800, 930 (canonical), 1,100, 1,250, 1,400 (ref. [1]). (b) Heat maps of group-averaged BIC ranks for models in which the sustained input component took the form of a linear up-ramp (left, for the DL condition) or a boxcar (right, for the FR condition). For each subject and condition, each of the 8 fitted models (Fig. 5a in main text) was assigned a rank, with 1 indicating the relative best fit and 8 the worst; thus a best possible score of 1 in the group-averaged ranks depicted here would indicate that the model under consideration (the up-ramp model for the DL condition, the boxcar model for the FR condition) provided the relative best fit for every single subject in the associated condition. Grey cells in the plots correspond to impulse response parameter combinations for which the group-level rank of the model in question (i.e. the rank assigned to the model after averaging across all subjects' BIC scores) was less than 1; thus, the up-ramp model provided the best group-level fit to the DL condition data for every tested combination of impulse response parameters, while the boxcar model provided the best group-level fit to the FR condition data for all but a small subset of impulse response function shapes that were narrow and peaked late.

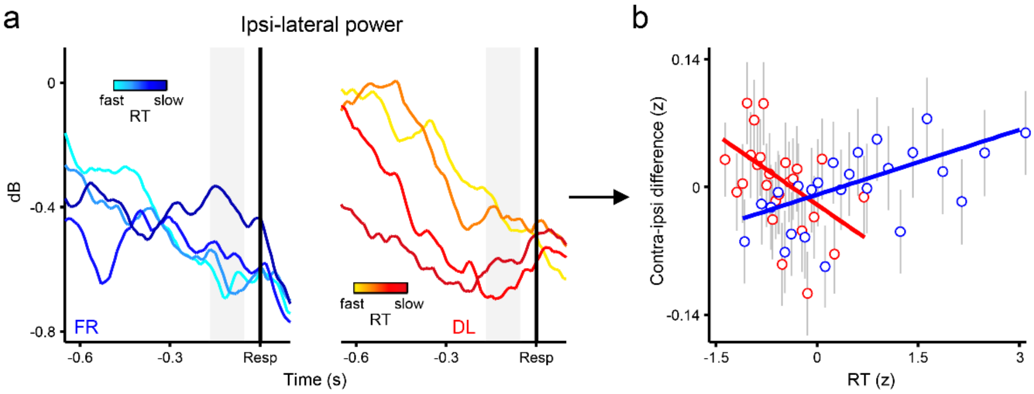

Supplementary Figure 6 | Time-dependency of pre-response ipsi-lateral 8-14Hz power. (a) Response-aligned *μ* (8-14Hz) signals ipsi-lateral to the executed response after sorting trials by RT into 4 equal-sized bins, separately for the FR and DL conditions (cold/hot colors, respectively). (b) Scatterplot illustrating the linear relationships between RT and pre-response ipsi-lateral *μ* power for each speed regime. Points and error bars are mean ±s.e.m. of data that were z-scored within subjects, pooled across subjects and grouped into 25 bins. Note that in both plots, *μ* signals are baselined at the single-trial level to better isolate the evoked, time-dependent change in the magnitude of the power decrease and eliminate potentially contaminating variation in baseline power. Hence, the main effect of speed emphasis that is due to the deadline-induced baseline offset in *μ* power (Fig. 2b of main text) is not apparent in either plot.

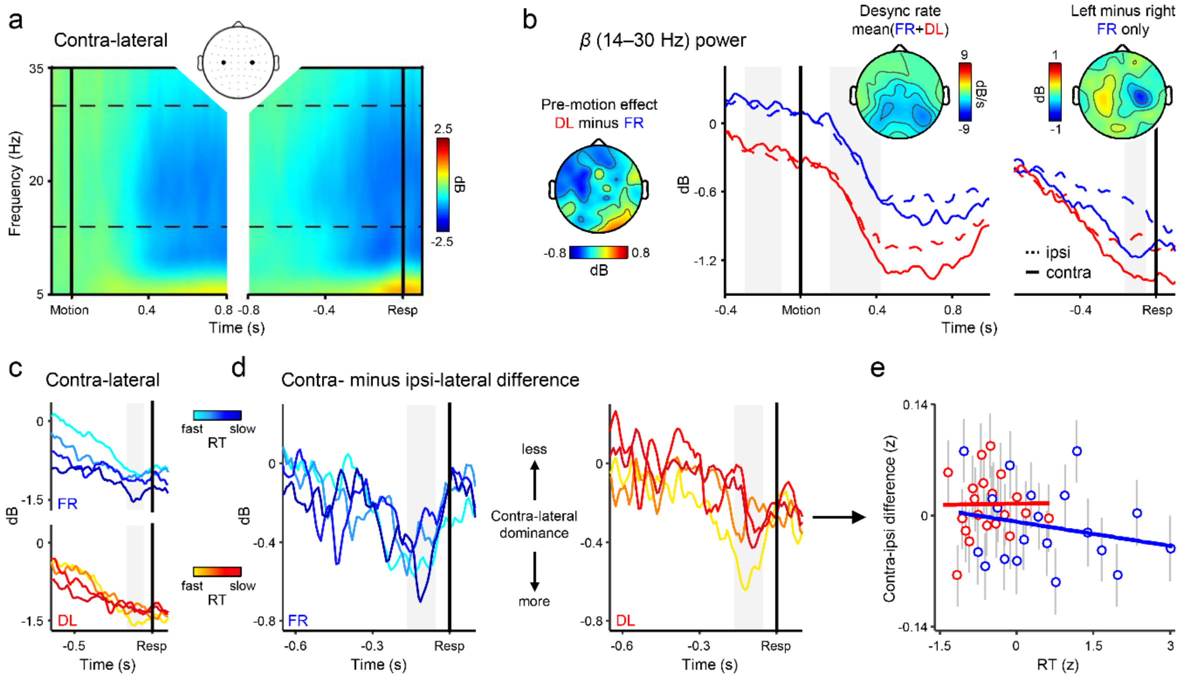

Supplementary Figure 7 | Effects of speed emphasis on 14-30Hz power. (a) Time-frequency plot of oscillatory power contra-lateral to the executed response, aligned to motion onset (left) and response (right) and relative to a pre-motion baseline. (b) Onset- and response-aligned β-band signals (14-30Hz), separated by speed regime and lateralization relative to the executed response. Topographies show the same information for β as corresponding topographies for μ (8-14Hz) power in Fig. 2 of the main text. Here, the contra-lateral β signals do not reach a stereotyped level of pre-response power across speed regimes, and pre-response power does not show any lateralization*speed regime interaction. (c) Response-aligned β waveforms contra-lateral to the executed response after sorting trials by RT into 4 equal-sized bins, separately for the FR and DL conditions (cold/hot colors, respectively). (d) Contra- minus ipsi-lateral difference waveforms, again after RT-sorting into 4 bins. There is no effect of RT on pre-response contra-lateral dominance in either speed regime. (e) Scatterplot illustrating the linear relationships between RT and the contra-/ipsi-lateral β difference for each speed regime. Points and error bars are mean ±s.e.m. of data that were z-scored within subjects, pooled across subjects and grouped into 20 bins; z-scoring was carried out across speed regimes.

Supplementary Tables

Supplementary Table 1 | Fit comparison of diffusion models with and without urgency

Urgency DDM						
Model #	u_0, λ, k	v	η	t_{er}	μ(BIC)	Σ(BIC)
1	free	fixed	none	fixed	21324	447814
2	free	free	none	fixed	21321	447732
3	free	fixed	none	free	21315	447608
4	free	free	none	free	21314	447602
5	free	fixed	fixed	fixed	21311	447535
6	free	free	fixed	fixed	21310	447502
7	free	fixed	fixed	free	21304*	447384*
8	free	free	fixed	free	21306	447420
Regular DDM						
Model #	a	v	η	t_{er}	μ(BIC)	Σ(BIC)
9	free	fixed	fixed	fixed	21517	451857
10	free	free	fixed	fixed	21515	451807
11	free	fixed	free	fixed	21520	451916
12	free	fixed	fixed	free	21490	451285
13	free	free	free	fixed	21521	451942
14	free	fixed	free	free	21493	451348
15	free	free	free	free	21495	451391

u_0 = static urgency component, λ/k = scale/shape parameters of logistic function determining time-dependent urgency component; v = drift rate, η = between-trial variability in drift rate, t_{er} = non-decision time, a = decision bound

* highlights best-fitting model, indicated by the lowest average and cumulative BIC values

Supplementary Table 2 | Fitted parameters from the best-fitting urgency diffusion model

Parameter	FR (mean ± SEM)	DL (mean ± SEM)	t	p
v	0.112 ± 0.007		-	-
η	0.063 ± 0.007		-	-
t_{er}	0.391 ± 0.019	0.355 ± 0.014	-1.87	0.076
u_0	0.872 ± 0.005	0.889 ± 0.007	2.19	0.041
λ	6.428 ± 0.679	18.697 ± 1.726	7.01	$<1x10^{-6}$
k	9.562 ± 1.155	0.861 ± 0.037	-7.59	$<1x10^{-6}$

t-scores and *p*-values derived from paired-samples *t*-tests comparing FR and DL parameter estimates.

Supplementary Methods

Task procedures

Unless otherwise stated below, stimuli were presented on a 47cm-wide LCD monitor operating at 60Hz and a resolution of 1680 X 1050 pixels. In all experiments, subjects were seated 60cm from the monitor and were instructed to minimize movement and eye-blinks during task performance. Testing was conducted under moderate-to-low levels of ambient light (≤18 cd/m^2 across experiments).

During task performance, subjects were instructed to maintain fixation on a centrally presented light-blue cross (occupying 0.36° of the visual angle). Coherent dot motion directions (leftward or rightward) were equiprobable and randomly selected across trials. The dots were white and 3 x 3 pixels in size, moved within a circle of 5° diameter at a speed of 5°/s and a density of 16.7 dots/degree^2/s, and were presented on a black background. Dots were assigned random positions for the first three frames of a trial and for each of these frames the dots were repositioned after two subsequent frames (i.e. dots in frame 1 were repositioned in frame 4, frame 2 dots were repositioned in frame 5, etc.). The new location of a dot was either random or in the dominant direction of motion on that trial. The probability with which each dot moved in this pre-determined direction is defined as coherence *c'*.

All subjects in all tasks completed initial practice and difficulty calibration routines prior to the main testing. In tasks 1 and 2, practice consisted of blocks of 20, 20 and 40 trials with progressively increasing difficulty (*c'*=0.8, 0.4 and 0.2, respectively); in tasks 3 and 4, it consisted of at least one block of 40 trials with *c'*=0.5 and additional blocks until performance accuracy was > 70%. Practice blocks were followed by a longer block (250 trials in the first, second and fourth experiments below, 200 trials in the third experiment) in which *c'*=[0, 0.1, 0.2, 0.4, 0.8] (each level of *c'* was assigned an equal number of trials and all trials were presented in random order). Once complete, the proportional rate diffusion model[2] was fit to the RT and accuracy data from this block in order to estimate each individual's psychometric function. The *c'* values corresponding to particular accuracy levels (see below) were then interpolated from this function and used in the main task blocks.

At the beginning of testing, subjects were explicitly informed about the duration of the response deadline that they would be performing under. In order to stabilize subjects' estimates of precisely when this deadline occurred, initial practice and difficulty calibration blocks were always performed under the same deadline that was employed during main testing (see below) and subjects were encouraged to intentionally miss the deadline several times during practice.

Deadline vs free response with EEG

The first version of the task was administered to 21 individuals (16 female; age 22.8±s.d.=2.6 years). They were paid a minimum of €15 for participating, in addition to a variable bonus that depended on task performance during all non-practice blocks. Stimuli were delivered on a 36cm-wide CRT monitor operating at 100Hz and 64-channel scalp EEG was recorded during task performance.

Subjects performed 8 blocks of 180 trials of the RDM task under two different conditions. In free response (FR) blocks, they were instructed to perform the task as accurately as possible and were not under any time pressure. Moreover, to mitigate any tendency to abandon deliberation on trials when a decision was taking a relatively long time to be made in this condition, subjects were instructed to try to reach the same level of confidence in their choice on every trial of FR blocks and to avoid spurious guessing. The reasoning behind this instruction was that the type of task instructions that are often provided to subjects on free response choice RT tasks ("be as fast and accurate as possible" or some variant thereof) are vague, open to individual differences in interpretation, and can be interpreted as encouraging time-dependency, which would in turn diminish the contrast between FR and DL conditions that was of critical interest here.

Correct responses were rewarded by adding 0.5¢ to the subject's total payment, while incorrect responses were punished by subtracting 0.5¢. To discourage occasional premature/anticipatory responses, RTs faster than 0.2s were punished by subtracting 0.5¢. In deadline (DL) blocks, subjects were again instructed to perform the task as accurately as possible but were now under a hard response deadline of 1.4s. The monetary reward scheme

from the FR condition was also in place in DL blocks, in addition to a heavy penalty of 5¢ for missing the deadline. Thus a wise strategy in the DL condition was to always execute a response before the deadline arrived, which was made clear to subjects prior to task performance.

FR and DL blocks were administered in a A-A-A-A-B-B-B-B design with the intention that a consecutive run of DL blocks would allow subjects to form more stable internal estimates of the precise timing of the deadline. The order of administration was counter-balanced across subjects, and 80 additional practice trials were administered after the fourth block and performed under the same speed emphasis instructions as the remaining four blocks. Subjects who performed FR blocks first also received 80 practice trials under this speed regime prior to starting their first block. c' was fixed across trials at a subject-specific level that corresponded to 75% accuracy under deadline (calibrated c' = 12.6 ± SD = 3.6; range = [8.1, 21.9]). Pilot testing indicated that this discrimination difficulty yielded at least some trials with RTs near the deadline and translated to below-ceiling performance in the FR condition.

To minimize post-decisional sensory evidence accumulation[3] and avoid sensory or feedback-related EEG transients at the time of response execution, coherent dot motion transitioned upon response execution to purely random motion (c'=0) for a period of 0.4s (subjects were made aware of this and instructed to ignore the post-response motion). Thereafter, the moving dots were replaced with a mask of stationary dots that were randomly distributed within the 5º circle and displayed until the start of the following trial. In addition, the color of the fixation cross changed for 0.75s after mask onset according to the accuracy of the preceding response: green if correct, red if incorrect. Lexical feedback in red font was provided when responses were quicker than 0.2s or when a response had not been made by the time of the deadline ("too fast" and "too slow", respectively). The response-to-stimulus interval (RSI), including random motion and feedback durations, was drawn from a uniform distribution with a range of 1.65 to 2.15s. A summary of money earned in the previous block and total money earned so far was provided in the inter-block interval.

Deadline vs free response with pupillometry

A separate cohort of 23 individuals (10 male; age=24.4±3.2 years) performed the RDM task under DL and FR conditions concurrent to pupillometry. They were paid a minimum of €10 for participating, in addition to a performance-dependent bonus.

Subjects performed the task with their forehead and chin positions secured by a table-mounted headrest, ensuring constant viewing distance and position throughout. Task parameters were the same as reported for task 1 (here, calibrated c' = 12.9±5.4; range = [5.6, 28.8]), with the following exceptions. Subjects performed 10 blocks of 90 trials that were administered in a A-B-A-B... design, with order counter-balanced across subjects. An alternating block design was favored in this case to avoid confounding effects of task condition with known effects of time-on-task on pupil measures (e.g.[4]). Additional practice blocks of 10 trials were administered prior to each experimental block in order to re-familiarize subjects with task instructions and re-establish the precise timing of the deadline on DL blocks. Several adjustments were also made to the task to avoid contaminating pupillary measures: The duration of purely random motion in the post-response period was extended to 1.75s to allow the dilation response to fully resolve before any gross change in the content of the visual display; no trial-by-trial feedback was provided so that the dilation response was not contaminated by feedback-related processes ("too fast" and "too slow" feedback was displayed when appropriate, but pupillary measures from these trials were not analyzed); and, the range of the RSI distribution was changed to [2.5, 3]s to negate contamination of the baseline period on trial n+1 by the dilation response on trial n.

Investigating time-dependency under mild speed pressure

To explore the generality of time-dependent urgency to task settings in which speed pressure is less stringent, we also report re-analyses of behavioral data from two previous studies from our lab. In both cases subjects performed the RDM task under a deadline of 1.5s, but were not penalized for missed deadlines and were simply instructed to perform the task 'as quickly and as accurately as possible'. This design and task instruction has been employed in human studies of perceptual decision-making that were not aimed at addressing the mechanistic basis of the speed/accuracy tradeoff (e.g.[5, 6]).

In the first of these two studies, originally described in Murphy et al.[6], subjects (n=26; 22 female; age=22.5±2.7 years) were given course credit or a gratuity of €15 for their participation. They were administered 5 blocks of 100 trials with a single subject-specific *c'* (32.3±11.6; range = [16.1, 54.9]) that corresponded to a mean accuracy of 85%. Upon response execution, the moving dots were immediately replaced with a mask of stationary dots and the fixation cross changed color for 0.7s to indicate the accuracy of the response (green=correct, light-red=incorrect). Red lexical feedback was displayed after anticipatory responses (RT<0.1s; "too fast") or missed deadlines ("too slow"). RSIs were drawn from a uniform distribution with range [5, 6.5]s.

In the second study, 21 individuals (7 male; age=23.0±3.0 years) were granted a minimum of €15 or 4 course credits for participating in addition to 0.5¢ for every correct response. They were administered 8 blocks of 160 trials with two levels of *c'* (12.3±5.6 and 25.4±11.4; ranges = [5.3, 29.2] and [11.0, 59.9], respectively) that corresponded to mean accuracies of 70% and 85% (equal number of trials per difficulty level per block, presented in random order). Motion onset was also preceded by probabilistic cues (neutral, valid, invalid; presented centrally for 0.8s) to manipulate subjects' *a priori* bias over the upcoming choice, but cue types were evenly distributed across both difficulty levels and we collapsed across them in the analysis of these data presented here (Fig. 7b). Coherent motion was presented for the full 1.5s irrespective of RT, followed by centrally presented visual feedback for 0.5s indicating either the money won on the preceding trial or "too slow" for missed deadlines. An additional jittered interval drawn from a uniform distribution with range [0.5, 1]s was presented before subsequent trial onset.

EEG acquisition and analysis

Eye-blinks and other noise transients were isolated and removed from the EEG via independent component analysis (ICA). Specifically, continuous data for each subject were concatenated across blocks, re-referenced to channel Fz, high-pass filtered to 1Hz and low-pass filtered up to 40Hz using a two-way least-squares FIR filter; noisy channels were identified by visual inspection of signal variance and removed; data were segmented into epochs from -0.4 to 1.6s relative to motion onset and baseline-corrected to the average of the entire epoch; epochs containing values that violated kurtosis and joint probability criteria (both ±4.5 s.d.[7]) were rejected; and, the remaining data were subjected to temporal ICA using the infomax algorithm. ICA weights were then back-projected to continuous, filtered EEG data (high-pass 0.1Hz, low-pass 40Hz). Next, ICs representing stereotyped artifactual activity such as eye blinks were identified by visual inspection and removed. Previously-identified noisy channels were then interpolated via spherical spline interpolation and the data were re-referenced to the common average. Data epochs were extracted on each trial from -0.6 to 1.8s relative to motion onset and -1.8 to 0.6s relative to response, and baseline-corrected to the 0.25s interval preceding motion onset. Subsequent epoch rejection employed a dynamic window with a fixed start time of -0.3s relative to motion onset and an end time of RT + 0.15s for each trial. For FR trials on which RT exceeded the time segments covered by the epoch bounds, artifact rejection was confined to data within the bounds. Epochs were rejected from all further analyses if any scalp channel exceeded ±60µV at any point within this trial-specific window. Lastly, all EEG data were converted to current source density[8] to increase spatial selectivity and minimize volume conduction.

For each electrode and each epoch, we calculated a time-frequency representation of the EEG power via complex Morlet wavelet convolution (4 frequency cycles per wavelet across each of 36 linear-spaced frequencies from 5-40 Hz, using the EEGLAB 'newtimef' function). The resulting power estimates were normalized by the decibel (dB) transform ($dB=10*\log_{10}[power/baseline]$). Unless otherwise specified, the baseline consisted of across-trial, across-condition and, for comparisons involving both contra- and ipsi-lateral signals, across-lateralization average power from -0.3 to -0.1s relative to motion onset. This averaged baseline preserved any condition- and lateralization-related differences in pre-stimulus power. The power of effector-specific μ oscillations, employed here as an index of motor preparation both contra- and ipsi-lateral to the executed response, was measured by averaging power between 8-14Hz[9], separately for electrodes C3 (left hemisphere) and C4 (right hemisphere). These electrodes were selected by inspection of the grand-average difference topography for leftward minus rightward responses (Fig. 2b, right inset).

We interrogated relationships between μ power and decision-making behavior via single-trial within-subjects regression. Unless otherwise stated, both DL and FR trials were first considered in the same multiple regression models and observed interactions were then decomposed via simpler condition- or lateralization-specific

regressions. To examine the effect of speed emphasis on pre-stimulus μ power for both contra- and ipsi-lateral effectors, we fit the following linear regression model to data from each subject:

$$Mu_{base} = \beta_0 + \beta_1 \times C + \beta_2 \times L + \beta_3 \times (C \times L) \quad (1)$$

where Mu_{base} represents μ power during the baseline period z-scored across condition and lateralization, C indicates speed emphasis condition (1=DL, 0=FR), L indicates lateralization (1=contralateral, 0=ipsilateral) and C X L represents the condition by lateralization interaction term. For all models, β_i are fitted regression coefficients. Topographic visualization of the effect of condition on pre-stimulus 8-14Hz power indicated that the effect was not restricted to motor regions but also extended over posterior scalp (Fig. 2b, left). To examine the relationship between motor-related μ and speed emphasis condition independent of this posterior effect, we fit an extended model:

$$Mu_{base} = \beta_0 + \beta_1 \times C + \beta_2 \times L + \beta_3 \times (C \times L) + \beta_4 \times Mu_{occ} \quad (2)$$

where the final term is a covariate containing z-scored pre-stimulus 8-14Hz power averaged over a selection of bi-lateral parieto-occipital channels (P5, P6, P7, P8, PO7, PO8).

The following model was constructed to examine the effect of speed emphasis condition on pre-response μ power:

$$Mu_{resp} = \beta_0 + \beta_1 \times C + \beta_2 \times L + \beta_3 \times (C \times L) \quad (3)$$

where Mu_{resp} is z-scored μ power during the pre-response measurement period, and all predictors are identical to those in Supplementary Equation (1). It is possible that condition-related effects yielded by this model are driven by the large difference in RTs between conditions – this is a concern particularly because, despite restricting our analysis to trials with RT>0.5s, the aforementioned stereotyped μ desynchronization immediately after stimulus onset may have still produced greater pre-response desynchronization for faster RTs, independent of any motor preparation effects. To mitigate this concern, we also fit the model in Supplementary Equation (3) to subsets of trials from each condition that were precisely matched for RT (Supplementary Fig. 1).

As a corollary to the model in Supplementary Equation (3), we also tested whether the level of contra-lateral μ desynchronization varied with response time in each speed emphasis regime. To do so, we fit simple linear regression models of the following form:

$$Mu_{resp} = \beta_0 + \beta_1 \times RT \quad (4)$$

to DL and FR trials separately, where RT indicates z-scored, log-transformed RT.

Next, we interrogated motor μ signals for evidence of a time-dependent influence on motor preparation that varied with speed pressure. Specifically, we examined whether the contra-/ipsi-lateral difference in pre-response μ desynchronization varied with RT and speed emphasis (see Methods section of main manuscript) using the following model:

$$Mu_{resp,c-i} = \beta_0 + \beta_1 \times C + \beta_2 \times RT + \beta_3 \times (C \times RT) \quad (5)$$

where $Mu_{resp,c\text{-}i}$ represents the contra- minus ipsi-lateral pre-response μ power z-scored across trials, C indicates speed emphasis condition, RT is z-scored, log-transformed RT, and C X RT is the condition by RT interaction. For this analysis, all μ signals were baseline-corrected at the single-trial level to eliminate trial-by-trial variation in pre-stimulus power and thus focus specifically on the evoked change in signal amplitude. However, the main effect of condition (β=0.095±0.028, t_{20}=3.6, p=0.003) and critical C X RT interaction (β=0.066±0.026, t_{20}=2.5, p=0.021; Fig. 2d,e) were also present using the same condition-, lateralization- and trial-averaged baseline employed in previous analyses. For completeness, we also fitted a version of the Supplementary Equation (5) model in which the pre-response amplitude of the ipsi-lateral μ signal alone was used as the dependent variable as opposed to the contra/ipsi difference (see Supplementary Fig. 7).

Pupillometric analysis

Eye-blinks and other noise transients were removed from pupillometric time series offline using a custom linear interpolation algorithm that restricted interpolation to periods of consecutive data loss (as registered by the Eyelink blink detection algorithm) that were shorter than 1s. Trials containing any remaining artifactual samples from -0.5s before motion onset to 2.5s post-response were excluded from analysis. As for the EEG analysis, trials with RTs<0.5s and >5s were also excluded for all pupil analyses with the exception of the pre-motion pupil trial matching CAF analysis depicted in Supplementary Fig. 1. Pupil time-series were low-pass filtered to 6Hz (fourth-order Butterworth) and z-scored using the mean and s.d. pupil diameter from all remaining clean samples across all blocks for a given subject, thus leaving condition-related differences in unbaselined pupil diameter intact.

The following within-subjects regression model was constructed to examine the main effect of speed emphasis condition on evoked pupil dilation and whether this effect interacted with RT:

$$Pupil_{resp} = \beta_0 + \beta_1 \times C + \beta_2 \times RT + \beta_3 \times (C \times RT) \tag{6}$$

where $Pupil_{resp}$ represents z-scored pupil dilation and all predictors are equivalent to those in Supplementary Equation (5).

Drift diffusion model fitting

We fit a number of standard and urgency DDMs to the behavioral data from the first cohort of subjects (Supplementary Table 1) and estimated parameters for each model and subject separately using maximum likelihood estimation. For each observed correct trial *c* and error trial *e* in each condition, we calculated the predicted defective probability densities $f_C(\mathrm{RT}_c|\theta)$ and $f_E(\mathrm{RT}_e|\theta)$ of observing those trials given a set of model parameters θ. Missed deadlines were also incorporated into the likelihood estimates by assigning them a probability P_M=1–$F(1.4|\ \theta)$, where $F(t|\theta)$ is the predicted cumulative probability for the DL condition at time *t*. The objective function to be minimized during fitting was:

$$L = -\sum_{c=1}^{n_C} \log f_C(RT_c|\theta) - \sum_{e=1}^{n_E} \log f_E(RT_e|\theta) - (n_M \times \log P_M) \tag{7}$$

where n_C is the total number of observed correct trials, n_E is the total number of observed error trials, and n_M is the number of observed misses. For fits of the standard DDM, expressions for $f_C(t|\theta)$, $f_E(t|\theta)$ and $F(t|\theta)$ were derived analytically using established methods described in Ratcliff & Tuerlinckx[10]. For the urgency DDM, we invoked a method for calculating first passagee time densities through continuously differentiable time-varying bounds that is based on the analysis of renewal equations[11]. For brevity we do not describe the method here, though useful summaries for decision scientists are given by Smith[12] and Zhang et al.[13]. Estimation of predicted densities using this approach can be much more time-consuming than the simple operations needed to analytically derive predictions for the standard DDM, particularly when the RT distributions to be fitted have long right tails (as in the FR condition here). To render the method suitable for lengthy parameter estimation routines, we estimated $f_C(t|\theta)$ and $f_E(t|\theta)$ at a temporal resolution of 0.02s and interpolated to sub-ms resolution via cubic spline interpolation. Testing against finer resolutions indicated that this approach resulted in little-to-no loss of fidelity for a realistic range of urgency signal shapes.

We adjusted the model parameters to optimize goodness-of-fit using particle swarm optimization[14] (PSO), setting wide bounds on all parameters and running 50 particles for 500 search iterations. We repeated this routine three times for each model variant and chose the best-fitting parameter set overall[15]. The relative goodness-of-fit of different model variants was assessed using the BIC, this time calculated as:

$$BIC = 2L + k \log n \tag{8}$$

where *k* is the number of free parameters and *n* is the total number of trials. As for the pupillometric analyses, Wilcoxon signed rank tests were used to compare the fit of two competing models.

We also leveraged the urgency DDM to approximate the optimal, reward-maximizing time-dependent urgency signals for the specific incentive scheme and deadline employed in studies 1 and 2. To do so, we chose a representative set of time-invariant parameters (taken as the across-subject mean DL estimates of v, η, t_{er} and u_0

from the best-fitting urgency DDM; Supplementary Table 2) and, for two closed-form functions, calculated the net expected reward given model predictions from a broad range of function-specific 'time-variant' parameters. The optimal urgency signal for each closed-form function was then identified by the parameter set that yielded the largest expected reward across the entire tested range. The closed-form functions in question were the logistic function of Equation 4 in the main text, and a simple linear function $u(t) = u_0+\beta t$. The urgency signals identified by this analysis (Supplementary Fig. 3) are only optimal within the limits of these closed-form functions, and as such do not directly correspond to the unconstrained, truly optimal decision policy given our task. However, given the flexibility of form inherent in the logistic function, and the similarity between the reward-maximizing signals identified here and those determined elsewhere via more sophisticated dynamic programming techniques[16], our reward-maximizing logistic urgency signals likely provide a reasonably good approximation of the truly optimal form of urgency for the representative set of time-invariant model parameters chosen.

To identify the reward-maximizing logistic urgency signal, we performed a grid search across 100 values of both λ (range = [0.5, 18]) and k (range = [0.7, 70]); for the linear function, we generated predictions for 300 values of the β parameter (range = [0.05, 3]). This process was repeated for each of 8 levels of endogenous uncertainty over the timing of the deadline. In accordance with previous work on the estimation of time intervals[17], timing uncertainty was defined as the coefficient of variation (CV = SD/mean) of a normal distribution of estimated deadlines with a mean centered on the true deadline of 1.4s. The CV values that we considered ranged from 0 to 0.14 in steps of 0.02; for each non-zero CV value, net expected reward for a given parameter set was estimated by generating model predictions and associated expected rewards for each of 51 nodes on the deadline distribution and numerically integrating across the expected reward associated with each node via Gaussian quadrature.

Leaky competing accumulator model fitting

We generated behavior from the adapted LCA model, as well as trial-by-trial activation time-series for both accumulator units, by Monte Carlo simulation with a step size of 0.01s. To enable examination of the effects of global gain modulation on the activation time-series in both the pre-stimulus and decisional periods, the simulation for each trial began 1s before motion onset and terminated at decision commitment. A baseline input (I_{base}) was fed into both input units during the pre-stimulus period and the network was allowed to stabilize, without noise, before motion onset. (Noise during the pre-stimulus period is not critical for the effects of interest here and was not modelled in order to avoid an additional free parameter.) In keeping with the best-fitting urgency DDM above, we also allowed the strength of motion stimulus input (I_1) to be normally distributed across trials (with s.d.=η) and incorporated a non-decision time parameter (t_{er}) that was allowed to vary across speed emphasis regimes.

Our intent with the LCA model was to demonstrate that a change in global gain state alone is capable of reproducing, in a qualitative sense, the key behavioral and neurophysiological effects of deadline-induced speed pressure, and as such we were not concerned with precise parameter estimation. Nonetheless, in its entirety the adapted model uses twelve free parameters to generate behavior for a single condition and is under-constrained. We therefore made the following assumptions to increase constraint on the fitting process: $I_{base} = 0.2$; $I_1 + I_2 = 1$ for all trials; $\theta = A$, corresponding to a transfer function with peak output that matched the level of the decision bound (simulations with smaller and larger values of θ produced qualitatively similar results); $\lambda > \alpha$, to ensure that the network stabilizes during the pre-stimulus period; $\alpha = \beta*1.5$, ensuring that recurrent excitation is always greater than lateral inhibition (a pre-condition for a gain-driven increase in competition between accumulators for slower RTs; see Discussion in main text); t_{er} for each condition was equal to the group-averaged t_{er} estimates from the best-fitting urgency DDM; and, g was fixed at 0.01 for all decision times across all FR trials (ensuring an approximately threshold-linear transfer function in this condition; Fig. 6b). We then proceeded to fit the six remaining free parameters (I_1, λ, α, σ, A, η) to the observed behavior from task 1 FR trials alone, pooled across all subjects. With wide search bounds on these parameters, the fitted α values tended to be low and the model in turn failed to produce competitive accumulation dynamics. We therefore imposed the final additional constraint that α was subject to a lower bound of 0.1.

The model was fit by running 10,000 simulations per parameter set and using quantile maximum likelihood estimation[18] (10 evenly-spaced quantiles per RT distribution) to minimize the negative log likelihood of the data

given the model predictions via the same PSO algorithm employed earlier. This procedure yielded the following parameter estimates: I_1=0.547, λ=0.201, α=0.110, σ=0.493, A=8.03, η=0.036. We then fit the behavior from DL trials by fixing these parameters at these values, but allowing g to assume a different baseline level and to change in magnitude with elapsed decision time. Specifically, we parameterized the within-trial time-course of g using the same logistic function employed for the urgency DDM [Equation (4) of main text], and chose the function parameters by manual exploration of the three-dimensional parameter space (final values: u_0=0.12, k=1.85, λ_{gain}=2.4). For the final parameter sets, we generated 100,000 simulations per condition to precisely evaluate model predictions.

Inspired by recent arguments that decision-makers do not perfectly accumulate sensory evidence but instead rely on an accumulation process with a short time constant (i.e. accumulation with a strong leak)[19, 20], we conducted additional LCA simulations in which the time constant of the accumulation process τ was fixed at a low level. Given the time-varying non-linearity in the transfer function in our adapted LCA model, the true time constant of accumulation is difficult to estimate. However, the effective time constant under low gain (where the transfer function is linear) can be approximated by $\tau = dt / (\lambda - \alpha - \beta)$, where dt is the simulation step size of 0.01s. Note that each accumulator is still subject to a lower bound of zero, so this equation only corresponds to the true time constant when the activations of both accumulators are greater than zero; otherwise the β term is eliminated from the equation. In our additional simulations, τ was fixed at 0.167s to correspond exactly with the time constant in ref. 19, while network gain was now allowed to vary over time in both the FR and DL conditions (again to accord with ref. 19; gain at decision time = 0 was still fixed at 0.01 in the FR condition). Aside from these changes, all other constraints and fitting procedures were identical to those described above. This approach yielded the following parameter estimates for the FR condition: I_1=0.550, λ=0.227, σ=0.552, A=6.64, η=0.023, k=10.35, λ_{gain}=6.80 (this combination of k and λ_{gain} corresponds to a flat gain time-course for most decision times, despite being allowed to take other forms; Fig. 6c, inset); and the following additional parameters for the DL condition: u_0=0.08, k=0.87, λ_{gain}=6.49.

References

1. de Gee JW, Knapen T, Donner TH. Decision-related pupil dilation reflects upcoming choice and individual bias. *Proc Natl Acad Sci USA* **111**, E618-625 (2014).

2. Palmer J, Huk AC, Shadlen MN. The effect of stimulus strength on the speed and accuracy of a perceptual decision. *J Vis* **5**, 376-404 (2005).

3. Murphy PR, Robertson IH, Harty S, O'Connell RG. Neural evidence accumulation persists after choice to inform metacognitive judgments. *eLife* **4**, (2015).

4. Murphy PR, Robertson IH, Balsters JH, O'Connell RG. Pupillometry and P3 index the locus coeruleus-noradrenergic arousal function in humans. *Psychophysiology* **48**, 1532-1543 (2011).

5. Mulder MJ, Wagenmakers E-J, Ratcliff R, Boekel W, Forstmann BU. Bias in the brain: a diffusion model analysis of prior probability and potential payoff. *J Neurosci* **32**, 2335-2343 (2012).

6. Murphy PR, Vandekerckhove J, Nieuwenhuis S. Pupil-linked arousal determines variability in perceptual decision making. *PLoS Comput Biol* **10**, e1003854 (2014).

7. Delorme A, Makeig S. EEGLAB: an open source toolbox for analysis of single-trial EEG dynamics including independent component analysis. *J Neurosci Methods* **134**, 9-21 (2004).

8. Kayser J, Tenke CE. Principal components analysis of Laplacian waveforms as a generic method for identifying ERP generator patterns: I. Evaluation with auditory oddball tasks. *Clin Neurophysiol* **117**, 348-368 (2006).

9. Gould IC, Nobre AC, Wyart V, Rushworth MF. Effects of decision variables and intraparietal stimulation on sensorimotor oscillatory activity in the human brain. *J Neurosci* **32**, 13805-13818 (2012).

10. Ratcliff R, Tuerlinckx F. Estimating parameters of the diffusion model: Approaches to dealing with contaminant reaction times and parameter variability. *Psychon Bull Rev* **9**, 438-481 (2002).

11. Buonocore A, Giorno V, Nobile A, Ricciardi L. On the two-boundary first-crossing-time problem for diffusion processes. *J Appl Probab* **27**, 102-114 (1990).

12. Smith PL. Stochastic dynamic models of response time and accuracy: A foundational primer. *J Math Psychol* **44**, 408-463 (2000).

13. Zhang S, Lee MD, Vandekerckhove J, Maris G, Wagenmakers EJ. Time-varying boundaries for diffusion models of decision making and response time. *Front Psychol* **5**, 1364 (2014).

14. Birge B. PSOt: A particle swarm optimization toolbox for matlab. In: *Proc. IEEE Swarm Intell. Symp*. IEEE Swarm Intelligence Symposium (2003).

15. Hawkins GE, Forstmann BU, Wagenmakers EJ, Ratcliff R, Brown SD. Revisiting the evidence for collapsing boundaries and urgency signals in perceptual decision-making. *J Neurosci* **35**, 2476-2484 (2015).

16. Frazier P, Yu AJ. Sequential hypothesis testing under stochastic deadlines. *Adv Neural Inf Process Syst* **20**, 465–472 (2008).

17. Karsilar H, Simen P, Papadakis S, Balci F. Speed accuracy trade-off under response deadlines. *Front Neurosci*, **8**, 248 (2014).

18. Heathcote A, Brown S, Mewhort DJ. Quantile maximum likelihood estimation of response time distributions. *Psychon Bull Rev* **9**, 394-401 (2002).

19. Carland MA, Marcos E, Thura D, Cisek P. Evidence against perfect integration of sensory information during perceptual decision making. *J Neurophysiol* **115**, 915-930 (2016).

20. Thura D, Beauregard-Racine J, Fradet CW, Cisek P. Decision making by urgency gating: theory and experimental support. *J Neurophysiol* **108**, 2912-2930 (2012).
